# Cytochrome P450 27C1 Level Dictates Lung Cancer Tumorigenicity and Sensitivity towards Multiple Anticancer Agents and Its Potential Interplay with the IGF-1R/Akt/p53 Signaling Pathway

**DOI:** 10.3390/ijms23147853

**Published:** 2022-07-16

**Authors:** Hai-Ying Mo, Qi-Yao Wei, Qiu-Hua Zhong, Xiao-Yun Zhao, Dan Guo, Jin Han, Wachiraporn Noracharttiyapot, Lydia Visser, Anke van den Berg, Yan-Ming Xu, Andy T. Y. Lau

**Affiliations:** 1 Laboratory of Cancer Biology and Epigenetics, Department of Cell Biology and Genetics, Shantou University Medical College, Shantou 515041, China; 16hymo@alumni.stu.edu.cn (H.-Y.M.); 19qywei@stu.edu.cn (Q.-Y.W.); 17qhzhong@stu.edu.cn (Q.-H.Z.); 18xyzhao@stu.edu.cn (X.-Y.Z.); 16jhan1@alumni.stu.edu.cn (J.H.); nora@stu.edu.cn (W.N.); 2 Department of Pathology and Medical Biology, University Medical Center Groningen, University of Groningen, 9700 RB Groningen, The Netherlands; l.visser@umcg.nl (L.V.); a.van.den.berg01@umcg.nl (A.v.d.B.); 3 Department of Pathology, Shantou University Medical College, Shantou 515041, China; dguo@stu.edu.cn

**Keywords:** CYP27C1, lung cancer, tumorigenesis, drug sensitivity, IGF-1R/Akt/p53 signaling

## Abstract

Cytochrome P450 enzymes (CYP450s) exert mighty catalytic actions in cellular metabolism and detoxication, which play pivotal roles in cell fate determination. Preliminary data shows differential expression levels of CYP27C1, one of the “orphan P450s” in human lung cancer cell lines. Here, we study the functions of CYP27C1 in lung cancer progression and drug endurance, and explore its potential to be a diagnostic and therapeutic target for lung cancer management. Quantitative real-time PCR and immunoblot assays were conducted to estimate the transcription and protein expression level of CYP27C1 in human lung cancer cell lines, which was relatively higher in A549 and H1975 cells, but was lower in H460 cells. Stable CYP27C1-knockdown A549 and H1975 cell lines were established, in which these cells showed enhancement in cell proliferation, colony formation, and migration. In addition, aberrant IGF-1R/Akt/p53 signal transduction was also detected in stable CYP27C1-knockdown human lung cancer cells, which exhibited greater tolerance towards the treatments of anticancer agents (including vinorelbine, picropodophyllin, pacritinib, and SKLB610). This work, for the first time, reveals that CYP27C1 impacts lung cancer cell development by participating in the regulation of the IGF-1R/Akt/p53 signaling pathway, and the level of CYP27C1 plays indispensable roles in dictating the cellular sensitivity towards multiple anticancer agents.

## 1. Introduction

Nowadays, 57 genes have been allocated to cytochrome P450 (CYP450) in the Human Genome Project [1], constituting an enzymatic family mainly serves as the terminal oxidase catalyzing monooxygenation reaction in the electron-transfer chains, and exerting indispensable function in detoxication and bioactivation of massive endogenous and exogenous molecules [2,3,4]. The major substrates of human CYP450s include, but are not limited to, steroids, fatty acids, eicosanoids, vitamins, xenobiotics, as well as unknown substrates, from which CYP450s can be roughly classified into six categories [5]. CYP450s participate in the biosynthesis, biotransformation, and catabolism of a wide variety of bioactive substances, e.g., steroid hormones [6], polyunsaturated fatty acids [3], eicosanoids [7], and fat-soluble vitamins [8,9,10]. Moreover, some CYP450s are known to be the primary enzymes responsible for phase I drug-metabolism, which is essential for biotransformation and elimination of drugs and toxicants. Generally, drugs are transformed to the less active form after CYP450-catalyzed oxidation reaction, and further undergo the metabolizing process [11]. On the other hand, CYP450s are required for the production of pharmacologically active form of some drugs, to obtain desired pharmacokinetics, avoid toxicity, or improve targeting ability [12]. Overall, CYP450s play pivotal roles in the maintenance of physiological homeostasis, and they are involved in the pathological courses of certain diseases [13].

It has been shown that genetic variants in CYP450 genes are correlated with high susceptibility to various types of malignant tumors, and can impact the therapeutic outcomes of CYP450-metabolizing anticancer drugs [14]. The existence of polymorphisms in carcinogen-metabolizing CYP450s can result in activated transformation and/or hindered elimination of carcinogens, which probably account for the increasing susceptibility of cancer in some individuals [15,16]. Since CYP450s are implicated in the pharmacokinetic process of a majority of anticancer drugs, more attention has been paid to the possible connection between CYP450 variants and inter-individual difference in therapeutic efficacy of some CYP450-metabolizing anticancer drugs, as well as the potential therapeutic values of CYP450 inhibitors [14].

Thirteen of the identified human CYP450s were termed as “orphan”, whose biological functions have not yet been clarified, including CYP2A7, 2S1, 2U1, 2W1, 3A43, 4A22, 4F11, 4F22, 4V2, 4X1, 4Z1, 20A1, and 27C1 [17,18]. Previously, to seek for the feasible target genes which might affect the progression and chemotherapeutic response in lung cancer, we detected the expression level of CYP450 genes in human lung cancer cell lines, and identified several differentially expressed CYP450 genes, one of which is *CYP27C1*. Human *CYP27C1* cDNA was first synthesized in *Escherichia coli* by polymerase cycling assembly in 2005, followed by obtaining the purified recombinant human CYP27C1 protein [19]. Despite the lack of thorough investigation of its biological function, human CYP27C1 was only reported as the enzyme catalyzing oxidation of all-trans-retinol (vitamin A1) to 3,4-dehydroretinol (vitamin A2) [20].

Here, to further explore the biological functions of CYP27C1 in human lung cancer cells, and evaluate the possibility of that being a target gene for lung cancer treatment, we established stable CYP27C1-knockdown human lung cancer cell lines. Alteration of proliferation, cell colony formation, migration, as well as the sensitivity towards different anticancer agents in stable CYP27C1-knocdown human lung cancer cells were further examined, which not only laid the foundations for revealing the mechanism of CYP27C1-associated lung cancer development, but also provided hints for identifying potential substrates of CYP27C1.

## 2. Results

### 2.1. CYP27C1 Is Differentially Expressed in Human Lung Cancer Cell Lines

**Figure 1:**
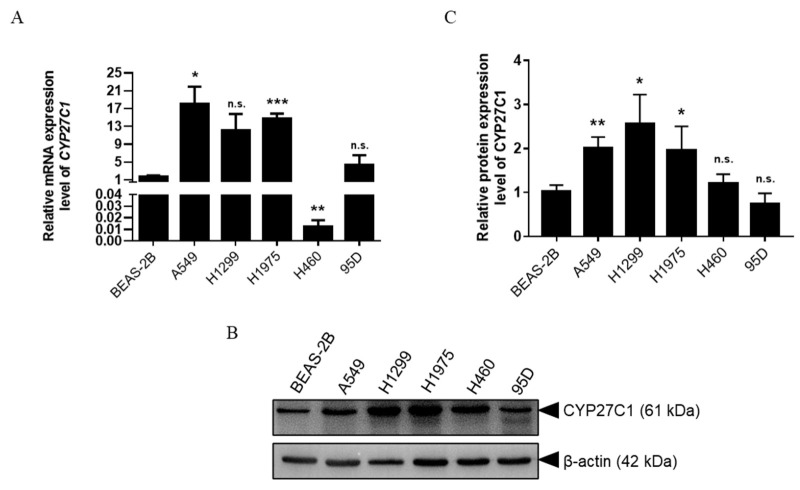
CYP27C1 expression in different human lung cancer cell lines. (**A**) Relative mRNA expression levels of CYP27C1 in human bronchial epithelial BEAS-2B cells, and human lung cancer cells were detected by real-time fluorescence quantitative PCR. Results comparing BEAS-2B with other cell lines were analyzed by unpaired two-tailed *T* test. *, *p* < 0.05; **, *p* < 0.01; ***, *p* < 0.001; n.s., *p* > 0.05. (**B**,**C**) Protein expression level of CYP27C1 in BEAS-2B, and human lung cancer cells were detected by immunoblot analysis. Grey scales of the bands were analyzed by Gel-Pro ANALYZER. Results were analyzed by unpaired two-tailed T test comparing BEAS-2B with other cell lines. *, *p* < 0.05; **, *p* < 0.01; n.s., *p* > 0.05.

To screen out the potential targets for the prevention, management, and prognostic assessment of lung cancer among CYP450 family members, we investigated the expression level of a range of CYP450 family members in human lung cell lines (including normal human bronchial epithelial BEAS-2B cells, and lung cancer cells). Among all the tested CYP450 family members, we noticed that CYP27C1, whose functions remain largely unexplored, was one of the differentially expressed genes (data not shown). Analysis of the expression level of CYP27C1 in different human lung cancer cell lines showed that the mRNA level of *CYP27C1* was significantly higher in A549 (*p* = 0.0276) and H1975 (*p* = 0.0007), and relatively lower in H460 (*p* = 0.0014), compared to that in BEAS-2B cells (Figure 1A). In addition, we found that the protein expression level of CYP27C1 was relatively higher in A549 (*p* = 0.0037), H1299 (*p* = 0.0177) and H1975 (*p* = 0.0431) in comparison with BEAS-2B cells (Figure 1B,C), indicating that the mRNA and protein expression level of CYP27C1 is aberrant in some human lung cancer cells.

### 2.2. Stable CYP27C1-Knockdown Enhances Lung Cancer Progression

**Figure 2:**
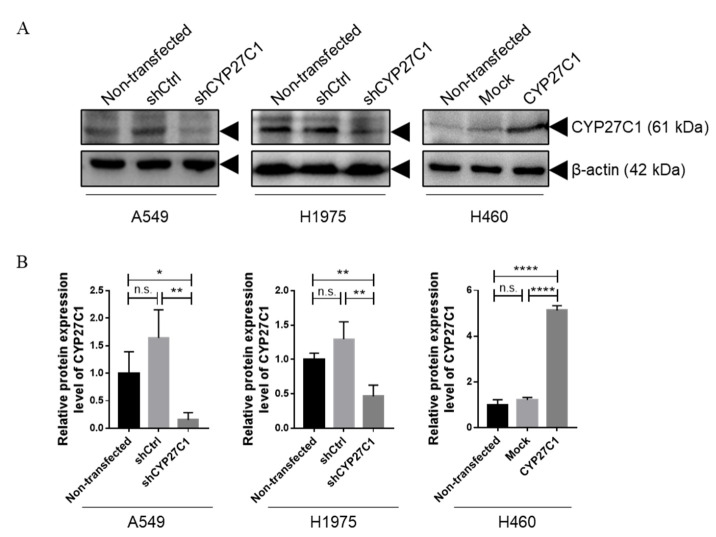
Construction of stable CYP27C1-knockdown or stable CYP27C1-overexpressed human lung cancer cell lines. (**A**) Protein level of stable CYP27C1-knockdown A549, H1975 cells, stable CYP27C1-overexpressd H460 cells, and the corresponding non-transfected control and vector control cells was detected by immunoblot analysis. (**B**) Grey scales of the bands were analyzed by Gel-Pro ANALYZER. Results were analyzed by unpaired two-tailed T test. Lines with blunt arrow indicated the comparison between different groups. *, *p* < 0.05; **, *p* < 0.01; ****, *p* < 0.0001 n.s., *p* > 0.05.

**Figure 3:**
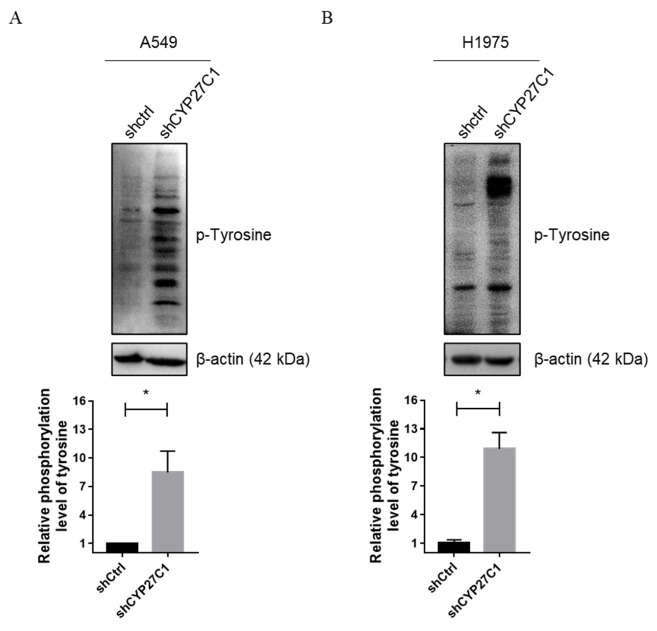
Stable CYP27C1-knockdown increases basal tyrosine phosphorylation level. Protein level of stable CYP27C1-knockdown A549 (**A**) stable CYP27C1-knockdown H1975 cells (**B**) and the corresponding control cells was detected by immunoblot analysis. Grey scales of the bands were measured by Gel-Pro ANALYZER. Results were analyzed by unpaired two-tailed T test. Lines with blunt arrow indicated the comparison between different groups. *, *p* < 0.05.

Studies found that the expression level and polymorphisms of cytochrome P450 family members are correlated with cancer susceptibility, development, and prognosis [14,21,22]. To further study the functions of CYP27C1 in human lung cancer, we first established stable CYP27C1-knockdown A549 and H1975 cell lines, and stable CYP27C1-overexpressed H460 cell line. As shown in Figure 2, CYP27C1 was significantly depleted in stable CYP27C1-knockdown A549 (vs. A549-shctrl, *p* = 0.0081) and H1975 (vs. H1975-shctrl *p* = 0.0087), while exogenous CYP27C1 was ectopically expressed in the stable CYP27C1-overexpressed H460 cell line (vs. H460-Mock, *p* < 0.0001). Interestingly, relatively higher basal tyrosine phosphorylation level was detected in stable CYP27C1-knockdown A549 (*p* = 0.0421) and H1975 cells (*p* = 0.0156), compared to that in control cells (Figure 3), suggesting that the inference of CYP27C1 expression can perturb cell signal transduction, and might further affect cellular activity, including tumorigenesis, proliferation, and metastasis.

#### 2.2.1. Stable CYP27C1-Knockdown Aggravates Colony Formation

**Figure 4:**
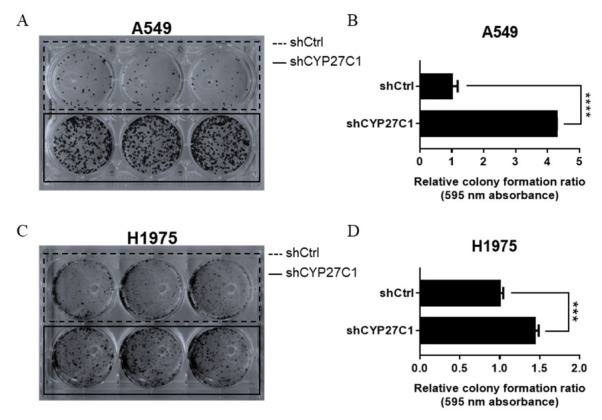
Stable CYP27C1-knockdown enhances colony formation ability of lung cancer cells. (**A**–**D**) A549-shCYP27C1, H1975-shCYP27C1, and the corresponding control cells were seeded in 6-well plates, respectively. Culture medium was changed in every 3–4 days, and cell colony formation was observed. After culturing for about 14 days, cell colonies were stained with crystal violet, and scanned. The absorbance of eluent was detected at 595 nm. Results were analyzed by unpaired two-tailed T test. ***, *p* < 0.001, ****, *p* < 0.0001.

Colony formation ability is one of the indicators of the tumorigenic capacity of cells, which can be affected by a wide range of factors. To test whether CYP27C1 participates in the tumorigenic process, we detected the colony formation ability of stable CYP27C1-knockdown cell lines, stable CYP27C1-overexpressed cell line, and the corresponding control cell lines by colony formation assay. The relative colony formation rate of stable CYP27C1-knockdown A549 cells was significantly higher compared to the control cells (*p* < 0.0001) (Figure 4A,B). The same pattern was also found in stable CYP27C1-knockdown H1975 cells and the corresponding control cells (*p* = 0.0004) (Figure 4C,D). However, the colony formation ability of stable CYP27C1-overexpressed H460 cells was similar to the control cells (*p* = 0.5916) (Supplementary Figure S1A). These results showed that knockdown of CYP27C1 led to greater amount of colony formation in both A549 and H1975 cells, which suggested that CYP27C1 hindered the colony formation process in some human lung cancer cell lines.

#### 2.2.2. Stable CYP27C1-Knockdown Facilitates Cell Proliferation

**Figure 5:**
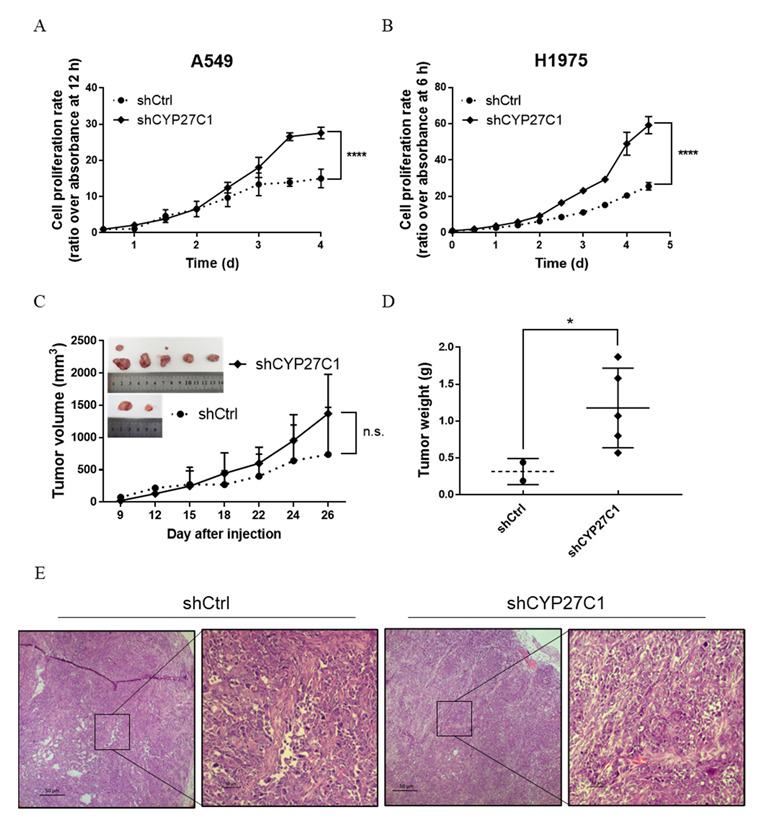
Stable CYP27C1-knockdown promotes human lung cancer cell proliferation in vitro, and in xenograft mice model. (**A**,**B**) Cell viability of A549-shCYP27C1, H1975-shCYP27C1, and the corresponding control cells was detected by MTS assay at different time points. Time-cell proliferation curves were plotted, and analyzed by two-way ANOVA. ****, *p* < 0.0001. (**C**) BALB/c nude mice (N = 10) were divided into two groups. H1975-shCYP27C1 cells were injected into the right armpit of the mice in experimental group, whereas H1975-shCtrl cells were injected into mice in control group. Tumor formed on the ninth day. Length (*l*) and width (*w*) of the tumor was measured on 12, 15, 18, 22, 24, and 26 days after injection. Tumor volume was calculated by formula: *V = (l×w^2^)/2*. Time-tumor volume curve was plotted, and analyzed by two-way ANOVA. n.s., *p* > 0.05. (**D**) Mice were sacrificed on the twenty-seventh day after injection. Mice were dissected, tumor weight was measured, and analyzed with Mann–Whitney test. *, *p* < 0.05. (**E**) Histology (hematoxylin-eosin staining) of representative xenograft tumor in nude mice model. Magnification: 50× (**panel 1,3**); 200× (**panel 2,4**).

In order to investigate whether CYP27C1 is involved in regulating cancer cell growth, we compared the proliferation rate of the stable CYP27C1-knockdown cells, stable CYP27C1-overexpressed cells, and the corresponding control cells. The results showed that the proliferation rate of stable CYP27C1-knockdown A549 (*p* < 0.0001) and H1975 (*p* < 0.0001) cells were both significantly higher than the corresponding control cells (Figure 5A,B), although there was no significant difference between the proliferation rate of stable CYP27C1-overexpressed H460 cells and the control cells (*p* = 0.9532) (Supplementary Figure S1B). To further verify the effects of CYP27C1 in cancer growth in vivo, we implanted the stable CYP27C1-knockdown H1975 cells and control cells into BALB/c nude mice and measured the tumor volume and tumor weight. The average tumor volume of stable CYP27C1-knockdown group was larger than that of control group from the eighteenth day since tumor cell injection, until the end of the experiment, though the growth curve of two groups showed no significant difference (*p* = 0.2956) (Figure 5C). Tumor weight of stable CYP27C1-knockdown group was significantly higher than that of control group (*p* = 0.0357) (Figure 5D). These results indicated that CYP27C1 depletion facilitates cell proliferation both in vitro and in vivo.

#### 2.2.3. Stable CYP27C1-Knockdown Promotes Cell Migration

**Figure 6:**
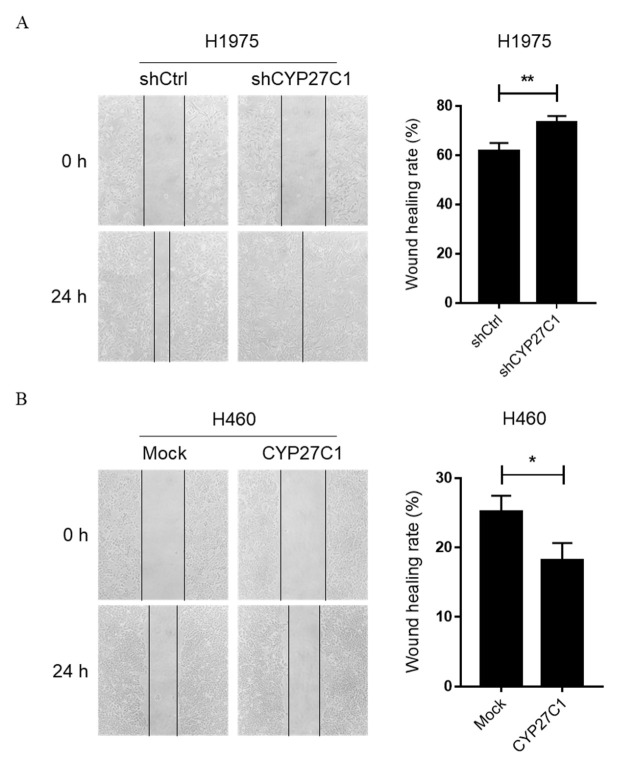
CYP27C1 expression affects ability of migration in lung cancer cells. (**A**,**B**) H1975-shCYP27C1, H460-CYP27C1, and the corresponding control cells were seeded in 12-well plates for 24 h before cell scratch assay. The wound areas were captured at different time points (0 h and 24 h). Scratching wound healing rate was analyzed by ImageJ software. Results were analyzed by unpaired two-tailed T test. *, *p* < 0.05, **, *p* < 0.01.

Additionally, we performed a cell scratch assay to detect the migration ability of stable CYP27C1-knockdown/overexpressed cells. Intriguingly, stable CYP27C1-knockdown H1975 cells showed enhanced migration ability (*p* = 0.0094) (Figure 6A), whereas the migration ability of stable CYP27C1-overexpressed H460 cells were significantly decreased (*p* = 0.0237) (Figure 6B), suggesting that CYP27C1 might be involved in the regulation of cell migration. However, the difference between cell migration ability of stable CYP27C1-knockdown A549 cells and the corresponding control cells was inconspicuous (*p* = 0.1937) (Supplementary Figure S2). 

#### 2.2.4. Stable CYP27C1-Knockdown Involves in Activation of IGF-1R/Akt/p53 Signaling Pathway

**Figure 7:**
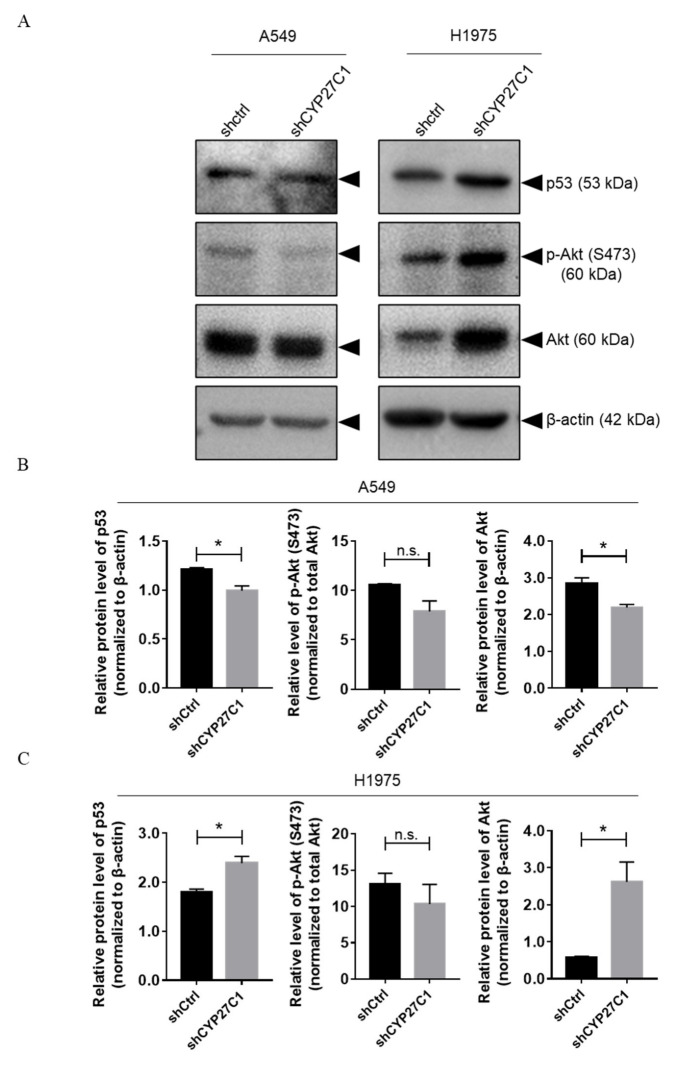
Stable CYP27C1-knockdown alters Akt-p53 signal transduction in human lung cancer cells. (**A**–**C**) A549-shCYP27C1, H1975-shCYP27C1, and the corresponding control cells were seeded in 6-well plate for 24 h before protein extraction and quantitation. Total Akt, phosphorylated Akt, p53, and β-actin were detected by immunoblot analysis using the corresponding antibodies. Grey scales of the bands were measured by Gel-Pro ANALYZER, and analyzed by unpaired two-tailed T test. *, *p* < 0.05, n.s., *p* > 0.05.

The IGF-1R/Akt/p53 signaling pathway is responsible for regulation of multiple cellular activity, whose disorder can lead to lung carcinoma and anticancer drug resistance [23,24]. We detected the phosphorylation level of Akt and expression level of p53 in stable CYP27C1-knockdown A549, H1975 cells. We found that the expression level of p53 (wildtype) was significantly lower in stable CYP27C1-knockdown A549 cells than that in control cells (*p* = 0.0267), whereas the expression level of p53 (R273H mutant) was elevated after stable CYP27C1-knockdown in H1975 cells (*p* = 0.0305) (Figure 7A, upper panels; Figure 7B,C, left panels). Unexpectedly, the phosphorylation level of Akt was relatively unchanged after stable CYP27C1-knockdown in both A549 and H1975 cells (Figure 7A, the second panels from the top; Figure 7B,C, middle panels). The level of total Akt significantly decreased in stable CYP27C1-knockdown A549 cells (*p* = 0.0333), whereas it markedly increased in stable CYP27C1-knockdown H1975 cells (*p* = 0.0329) (Figure 7A, the third panels from the top; Figure 7B,C, right panels). The above evidence showed that the inference of the expression of CYP27C1 can cause the alteration of the expression of p53 (wildtype and R273H mutant) and Akt, which is further involved in tumorigenesis, and the progression of lung cancer by promoting cell proliferation and migration. In other words, the regulation effects of CYP27C1 on cell colony formation, proliferation, and migration could be exerted via the IGF-1R/Akt/p53 signaling pathway.

### 2.3. IC_50_ Values of Anticancer Agents in Human Lung Cancer Cells

Given that the expression level of some CYP450 family members can influence certain cellular activities involved in cell proliferation and drug sensitivity, we detected and compared the sensitivity of four anticancer agents, including one approved drug for non-small cell lung cancer treatment (vinorelbine), and three protein kinase inhibitors (picropodophyllin, pacritinib, and SKLB610) in four human lung cancer cell lines (Supplementary Figure S4). IC_50_ values are shown in Table 1. 

Vinorelbine is one of the alkaloid chemotherapeutic drugs with curative effects for non-small cell lung cancer patients [25,26], which promotes cell death mainly by disturbing the microtubule dynamics [25], and whose metabolism is mainly conducted in the liver, and mainly catalyzed by CYP3A4 [27]. Among four human lung cancer cell lines, H1975 and H1299 showed greater resistance to vinorelbine treatment (Supplementary Figure S4E,M), whereas A549 was relatively more sensitive to vinorelbine, comparing to the other three cell lines (Table 1).

Picropodophyllin (PPP) is a selective insulin-like growth factor-1 receptor (IGF-1R) inhibitor that can block the phosphorylation of IGF-1R, Akt, and ERK1/2 stimulated by IGF-1 [28]. The IC_50_ values of PPP in A549, H1975, H460, and H1299 cells were low, signifying that PPP has great potency inhibiting the viability of all these human lung cancer cells. 

Pacritinib selectively inhibits Janus kinase 2 (JAK2) (wild type, and V617F mutant), and Fms-like tyrosine kinase-3 (FLT3) (wild type and D835Y mutant), disturbs cell cycle, and induces cell apoptosis [29]. Among four human lung cancer cell lines, H1975 cells showed the highest sensitivity towards pacritinib, whereas the inhibitory effect on H460 cells was the dullest.

SKLB610 is an effective tyrosine kinase inhibitor which can target vascular endothelial growth factor receptor 2 (VEGFR2), FGFR2 (fibroblast growth factor receptor 2), and platelet-derived growth factor receptors (PDGFR) [30]. SKLB610 treatment was most potent against the proliferation of A549 cells, whereas it showed the weakest inhibitory effect on H1975 cell growth.

### 2.4. CYP27C1-Knockdown Attenuates Anticancer Potency of Protein Kinase Inhibitors

Most protein kinase inhibitors are small molecules which are designed for inhibiting specific protein kinase receptors. High specificity and potency are prior criteria for a practicable protein kinase inhibitor; in the meantime, resistance is an inevitable challenge for most potential anticancer protein kinase inhibitors. As promising protein kinase inhibitors against multiple types of malignancies, PPP, pacritinib, and SKLB610 showed various degrees of inhibitory effects towards human lung cancer cells (Supplementary Figure S4, Table 1).

**Figure 8:**
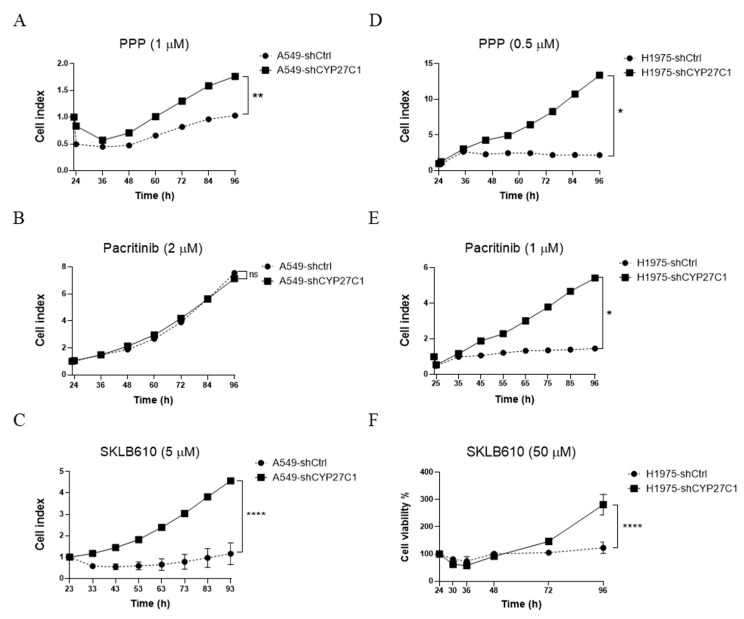
CYP27C1-knockdown attenuates anticancer potency of protein kinase inhibitors. (**A**–**E**) A549-shCYP27C1, H1975-shCYP27C1, and the corresponding control cells were treated with indicated concentration of protein kinase inhibitors (picropodophyllin, pacritinib, and SKLB610). Real-time cell index reflecting cell viability were detected by RTCA-S16 System. Results were analyzed by two-way ANOVA. ****, *p* <0.0001, **, *p* <0.01, *, *p* < 0.05, n.s., *p* > 0.05. (**F**) Stable CYP27C1-knockdown H1975 cells and the corresponding control cells were treated with indicated concentrations of SKLB610, cell viability was determined by MTS assay at different time point, and analyzed by two-way ANOVA. ****, *p* <0.0001.

To further explore the relationship between CYP27C1 expression level and potency of protein kinase inhibitors, as well as their resistance in human lung cancer cells, we employed stable CYP27C1-knockdown A549, H1975 cells, and detected cell viability with the treatment of each inhibitor. Surprisingly, we found that the treatment of PPP (1 μM) induced cytotoxicity in stable CYP27C1-knockdown A549 cells and control cells after 12 h. Both cell lines were released from the inhibitory effects of PPP after treating for 24 h, and initiated cell proliferation. However, stable CYP27C1-knockdown A549 cells showed more rapid cell growth compared to the control cells (Figure 8A). Mighty cell proliferation (cell index increased to 13 after treating for 72 h) appeared in CYP27C1-knockdown H1975 cells with the treatment of PPP (0.5 μM), whereas the cell viability of control cells was significantly inhibited (cell index was 2.1 after treating for 72 h) (Figure 8D).

After treating pacritinib (1 μM) for 72 h, the cell index of stable CYP27C1-knockdown H1975 cells steadily increased to 5.4, whereas the cell index of the control cells was less than 1.5 (Figure 8E), indicating that interference of the expression of CYP27C1 affects the inhibitory effect of pacritinib toward cell proliferation. However, the proliferation of both stable CYP27C1-knockdown A549 cells and the corresponding control cells were hardly inhibited by the pacritinib (2 μM) treatment (Figure 8B).

With the treatment of SKLB610, a cell index of A549 control cells decreased to 0.55 in 43 h, and gradually recovered to 1.2 in 93 h, whereas stable CYP27C1-knockdown A549 continuously grew until the cell index reached 4.6 in 93 h (Figure 8C). Different from that in stable transfected A549 cells, both CYP27C1-knockdown H1975 cells and control cells showed slightly declining cell proliferation rates 36 h after SKLB610 treatment, and recovered after 48 h. However, the proliferation rate of stable CYP27C1-knockdown H1975 cells reached 281% after treating for 96 h, whereas that of the control cells was 123% (Figure 8F), suggesting that CYP27C1 expression is critical for the function of SKLB610 restraining cell proliferation. The above results showed that inference of the expression of CYP27C1 could attenuate the inhibitory effects of protein kinase inhibitors, PPP, pacritinib, and SKLB610, in human lung cancer cells.

### 2.5. CYP27C1 Impacts Inhibitory Effect of Picropodophyllin via IGF-1R/Akt/p53 Signaling Pathway

The IC_50_ values of PPP are considerably low compared to that of other applied protein kinase inhibitors (Table 1), suggesting that PPP processes the highest potency inhibiting human lung cancer cell viability among three protein kinase inhibitors. However, we found that CYP27C1-knockdown significantly impairs the potency of PPP (Figure 8A,D). Therefore, to further study the role of CYP27C1 in PPP sensitivity, we detected the phosphorylation level of Akt and the expression level of p53 in stable CYP27C1-knockdown human lung cancer cells with PPP treatment.

**Figure 9:**
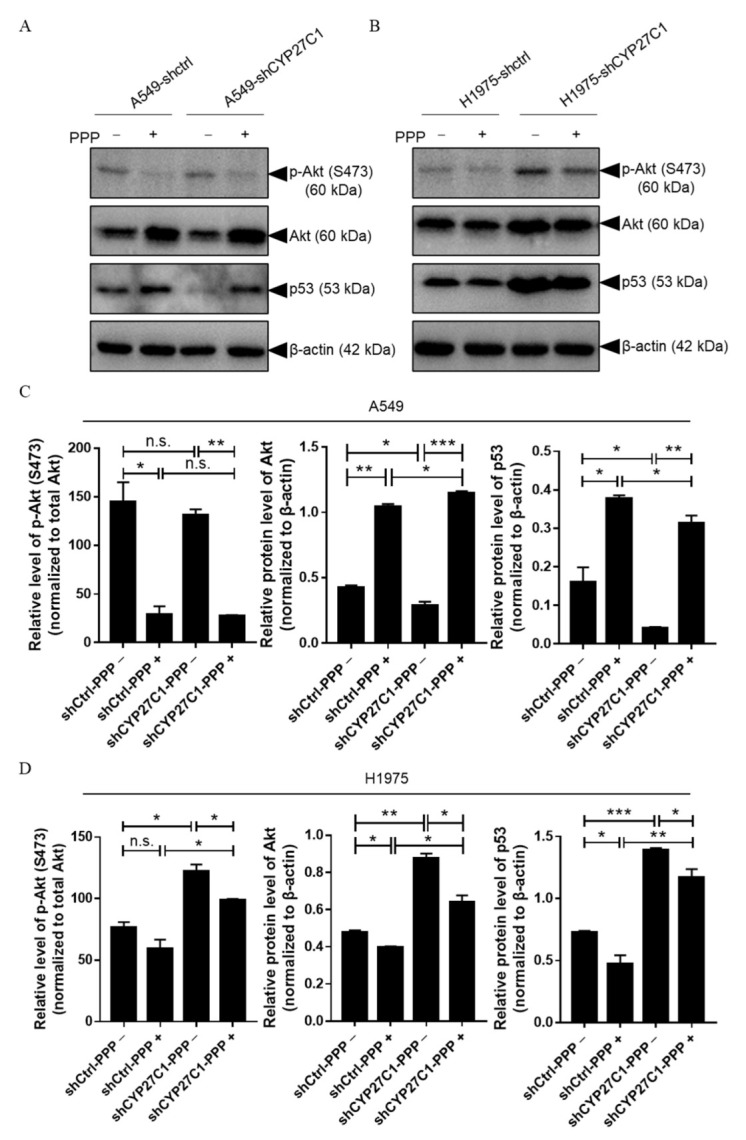
CYP27C1 impacts inhibitory effect of picropodophyllin via IGF-1R/AKT/p53 signaling pathway. (**A**,**B**) A549-shCYP27C1, H1975-shCYP27C1, and the corresponding control cells were seeded in 6-well plate for 24 h before treatment of PPP (100 μM, 24 h). Protein samples were applied to perform immunoblot analysis after extraction and quantitation. Total Akt, phosphorylated Akt, p53, and β-actin were detected by immunoblot analysis using the corresponding antibodies. (**C**,**D**) Grey scales of the bands were measured by Gel-Pro ANALYZER, and analyzed by unpaired two-tailed T test. *, *p* < 0.05, **, *p* < 0.01, ***, *p* < 0.001, n.s., *p* > 0.05.

We found that the phosphorylated levels of Akt significantly decreased in both stable CYP27C1-knockdown A549 and the corresponding control cells upon the PPP treatment (Figure 9C, Panel 1, Column 1 vs. Column 2, *p* = 0.0170; Column 3 vs. Column 4, *p* = 0.0014), whereas the level of phosphorylated Akt in stable CYP27C1-knockdown A549 and the corresponding control cells was similar, even with the treatment of PPP (Figure 9C, Panel 1, Column 1 vs. Column 3, *p* = 0.4539; Column 2 vs. Column 4, *p* = 0.8923). Interestingly, PPP treatment caused a steep increase of total Akt in both stable CYP27C1-knockdown A549 and control cells (Figure 9C, Panel 2, Column 1 vs. Column 2, *p* = 0.0010; Column 3 vs. Column 4, *p* = 0.0008). In the absence of PPP treatment, the level of total Akt was significantly downregulated in stable CYP27C1-knockdown A549 cells compared to the control cells (Figure 9C, Panel 2, Column 1 vs. Column 3, *p* = 0.0307), whereas PPP treatment caused a greater elevation of total Akt expression in stable CYP27C1-knockdown A549 cells (Figure 9C, Panel 2, Column 1 vs. Column 3, *p* = 0.0354). Without the treatment of PPP, the expression level of p53 (wildtype) declined in stable CYP27C1-knockdown A549 cells compared with the control cells (Figure 9C, Panel 3, Column 1 vs. Column 3, *p* = 0.0489), which is consistent with the previous result (Figure 7B, Panel 3). Notably, the expression level of p53 (wildtype) markedly upregulated in both stable CYP27C1-knockdown A549 cells and the control cells after PPP treatment (Figure 9C, Panel 3, Column 1 vs. Column 2, *p* = 0.0158; Column 3 vs. Column 4, *p* = 0.0026), whereas the control cells showed greater enhancement of p53 (wildtype) expression in the presence of PPP, compared to stable CYP27C1-knockdown A549 cells (Figure 9C, Panel 3, Column 2 vs. Column 4, *p* = 0.0184).

On the other hand, stable CYP27C1-knockdown H1975 cells, which harbor mutated p53 (R273H), responded differently from stable CYP27C1-knockdown A549 cells when enduring PPP treatment. For example, the phosphorylation level of Akt in stable CYP27C1-knockdown H1975 cells was relatively higher than that in control cells, whether or not PPP was added (Figure 9D, Panel 1, Column 1 vs. Column 3, *p* = 0.0117; Column 2 vs. Column 4, *p* = 0.0184). In the meantime, the amounts of total Akt in stable CYP27C1-knockdown H1975 cells were also distinctly greater than that in the control cells (Figure 9D, Panel 2, Column 1 vs. Column 3, *p* = 0.0026; Column 2 vs. Column 4, *p* = 0.0126). Different from the response in stable CYP27C1-knockdown A549 cells, dampened expression of total Akt exhibited in both stable CYP27C1-knockdown H1975 cells and the control cells upon PPP treatment (Figure 9D, Panel 2, Column 1 vs. Column 2, *p* = 0.0132; Column 3 vs. Column 4, *p* = 0.0213) were detected. Similar to the effects of PPP on the level of total Akt, the level of p53 (R273H) was downregulated by PPP treatment (Figure 9D, Panel 3, Column 1 vs. Column 2, *p* = 0.0368; Column 3 vs. Column 4, *p* = 0.0471). Remarkably, in stable CYP27C1-knockdown H1975 cells, the level of p53 (R273H) was constantly higher than that in the control cells, even with PPP treatment (Figure 9D, Panel 3, Column 1 vs. Column 3, *p* = 0.0005; Column 2 vs. Column 4, *p* = 0.0096).

Taken together, we found that the treatment of PPP impaired the Akt-p53 signal transduction not only by inhibiting the phosphorylation of Akt, but also by regulating the protein level of total Akt and p53. Cells harboring a different type of p53 (wildtype or R273H) reacted differently upon PPP treatment, especially with regards to Akt and p53 expression. In particular, with PPP treatment, the level of Akt and p53-wildtype was upregulated in stable CYP27C1-knockdown A549 cells, whereas p53-R273H declined in stable CYP27C1-knockdown H1975 cells, compared to the corresponding control cells. Despite the differential changes in these cells when encountered with PPP treatment, they all suggest that stable CYP27C1-knockdown impeded the inhibitory effects of PPP by elevating the level of Akt, and altering the level of p53, in which wildtype was diminished, whereas R273H mutant increased.

### 2.6. CYP27C1-Knockdown Impairs Anticancer Effect of Vinorelbine

**Figure 10:**
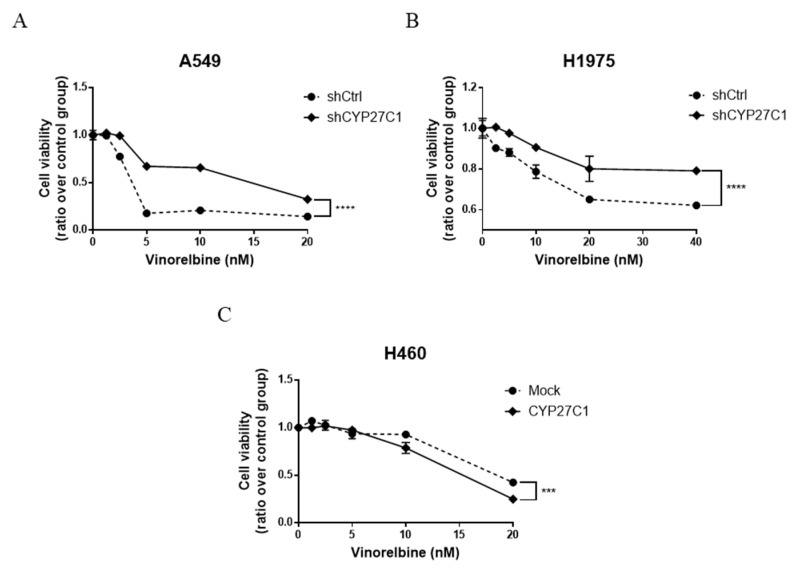
CYP27C1-knockdown impairs anticancer effect of vinorelbine. (**A**–**C**) A549-shCYP27C1, H1975-shCYP27C1, H460-CYP27C1, and the corresponding control cells were seeded in 24-well plates, respectively. After seeding for 24 h, cell medium was changed into that containing different concentrations of vinorelbine (0, 1.25, 2.5, 5, 10, 20 nM or 0, 2.5, 5, 10, 20, 40 nM). After treatment for 72 h, NBB assays were performed to detect cell viability. Results were analyzed by two-way ANOVA. ****, *p* < 0.0001, ***, *p* < 0.001.

To further investigate whether the expression level of CYP27C1 contributes to the alteration of vinorelbine potency in human lung cancer cells, we tested the sensitivity of stable CYP27C1-knockdown cells and stable CYP27C1-overexpressed cells toward vinorelbine. We found that the inhibitory effects of vinorelbine on cell growth of both stable CYP27C1-knockdown A549 and H1975 cells were significantly milder than that of the corresponding control cells (*p* < 0.0001) (Figure 10A,B), whereas stable CYP27C1-overexpressed H460 cells were more sensitive to vinorelbine compared to the control cells (*p* = 0.0006) (Figure 10C). These results indicated that CYP27C1 expression sustained the cytotoxic effects of vinorelbine.

## 3. Discussion

Most of the CYP450 family members implement their functions in biotransformation and metabolism of both endogenous and exogenous substances. Generally, drug metabolism via the enzymatic reaction catalyzed by CYP450 family members is the first step during the drug elimination process. Hence, the expression level of a specific CYP450 family member could impact the accumulation of drugs and further interfere with the therapeutic effects in the human body, which might affect the sensitivity of the tumor cells towards anticancer drugs. Moreover, some CYP450 family members are responsible for bioactivation or detoxification of certain types of chemical carcinogens by catalyzing metabolic reactions. Therefore, the expression level and activities of those related CYP450 enzymes are considered to be important factors that determinate the susceptibility of carcinogen-induced cancers [15,16,31]. To identify the molecules which are potentially involved in regulation of tumorigenesis, cancer progression, and sensitivity toward different anticancer drugs, we detected the expression level of multiple CYP450 family members in human lung cell lines. Accidently, we found a disparity of expression levels of CYP27C1 in different human lung cancer cell lines, implicating that CYP27C1 is probably involved in lung cancer development. Furthermore, the discrepancy of IC_50_ values of several anticancer agents in different human lung cancer cell lines aroused our interests in the relationship between CYP27C1 expression levels and anticancer drug sensitivity in human lung cancer.

Human CYP27C1 is one of the “orphan CYP450s” with an unclear biological function [17,18], which was first artificially expressed in *Escherichia coli* and briefly characterized when compared with other CYP27 subfamily members (CYP27A1 and CYP27B1) [19]. Subsequent studies demonstrated that CYP27C1 was the desaturase converting vitamin A_1_ (all trans-retinol) to vitamin A_2_ (all trans-3,4-dehydroretinol) in human skin [20,32]. Despite lack of evidence showing direct connection between the expression level of CYP27C1 and lung cancer, links between other CYP450 family members and carcinogenesis have been reported. Previous studies showed that silencing of *cyp1b1* in mice inhibits the metabolism of 7,12-dimethylbenz[*a*]anthracene (DMBA), and reduced the incidence rate of DMBA-induced malignancies [33]. Additionally, aberrantly abundant expression of CYP2J2, an epoxygenase, were detected in a series of human cancer cell lines [34], and its inhibition by selective inhibitor could induce apoptosis in human cancer cells [35].

Here, we intended to investigate whether there are latent connections between the expression level of CYP27C1 and lung cancer development. Thus, we chose A549 and H1975, which showed relatively higher gene and protein expression levels of CYP27C1, to establish stable CYP27C1-knockdown cell lines, and we employed H460 with relatively less CYP27C1 gene and protein expression to acquire a stable CYP27C1-overexpressed H460 cell line, followed by cellular activity assessments, including cell growth, colony formation, and sensitivity toward anticancer agents. Unexpectedly, we found that instead of restraining tumor progression, silencing endogenous CYP27C1 expression facilitated tumor cell proliferation and colony formation, and also aggravated tumor burden in nude mice models, indicating that CYP27C1 might act as a negative regulator participating in lung cancer cell proliferation. A similar phenomenon mediated by another CYP450 family member, CYP1A2, has been observed in hepatocellular carcinoma, in which CYP1A2 catalyzes metabolic transformation from 17β-estradiol to 2-methoxyestradiol, thus markedly inhibiting proliferation and triggering apoptosis [36]. Resemblance in cell behavior were observed in stable CYP27C1-knockdown A549 and H1975 cells, whereas it seemed that the Akt-p53 signaling pathway reacted differently upon CYP27C1 depletion in these two cell lines. One of the reasons for the discrepancy could be the mutation of p53 (R273H) in H1975 cells, which exert the opposite function to wildtype p53 harbored by A549 cells. Akt is the upstream regulator of p53 which alters the level and function of p53; in turn, p53 can affect the status of Akt through positive or negative feedback [23,37]. CYP27C1 might participate in cell behavior regulation via these feedback loops, which need to be further studied. Interestingly, we found that the migration ability of stable CYP27C1-overexpressed H460 cells significantly decreased compared to that in the corresponding control cells. In the meantime, in this study, we revealed that the expression level of CYP27C1 could affect the level of p53 and Akt, and might further participate in the regulation of cell migration. It is reasonable to hypothesize that the overexpression of CYP27C1 can upregulate the level of p53 (wildtype) in H460 cells, which may enhance the activation of downstream genes that suppress cell migration (e.g., CD82, PCDH7, and CXCR4), and further hinder cell migration [38]. However, the specific roles of CYP27C1 in the IGF-1R/Akt/p53 signaling pathway remain unclear.

The only reported substrate of CYP27C1 is vitamin A_1_, yet, the function of its metabolite, vitamin A_2_, remains largely unknown in the field of oncology. Although Vahlquist et al. found that the concentrations of vitamin A_2_ were elevated in human squamous cell carcinoma and keratoacanthoma compared to the healthy control epidermis, the physiologic role of vitamin A_2_ in skin cancer development has not yet been confirmed [39]. In this study, we found that modulation of the expression of CYP27C1 in human lung cancer cell lines affects their sensitivity towards vinorelbine, which is a front-line anticancer drug. Though it has been reported that vinorelbine is mainly metabolized by CYP3A4 in human liver microsomes [40], the catalytic activity of CYP27C1 on vinorelbine has not yet been investigated. By analyzing the structure of vinorelbine, we noticed that it could be desaturated at the 5-, 19-, and 2′-, 18′-position, similar with that in vitamin A_1_. Therefore, the possibility of vinorelbine being a novel substrate of CYP27C1, and the mechanism interpreting why CYP27C1 has impact on sensitivity of human lung cancer cells towards vinorelbine, could be our focus in further studies.

Protein kinase inhibitors are designed to precisely target the receptors of specific protein kinases, thereby suppressing tumor cell growth by impeding the transduction of the corresponding signaling pathway. Despite the inevitable resistance upon the administration of the majority of commercial protein kinase inhibitors, they are considered to be promising weapons for prolonging overall survival of cancer patients, owing to their remarkable potency against targeting kinase receptors and the downstream signaling [41,42]. CYP450 family members are responsible for the pharmacokinetic process of most commercial clinical protein kinase inhibitors [43,44], in other words, regulating their accumulation and clearance. Some CYP450 family members participate in particular cell signaling pathways affecting the progression of neoplastic transformation [45,46,47,48,49]. Therefore, to investigate the role of CYP27C1 in the anticancer mechanism of different types of protein kinase inhibitors, three agents were employed in this study, which target different cell signaling pathways. Strikingly, all the inhibitors partially lost their cytotoxicity in stable CYP27C1-knockdown human lung cancer cells. On the other hand, aberrantly active cell signal transduction was detected in stable CYP27C1-knockdown human lung cancer cells, suggesting that CYP27C1 might exert a function which is similar to a tumor suppressor in cell signal transduction, whose knockdown could overcome the inhibitory effects of protein kinase inhibitors. Therefore, uplifting the expression level or the catalytic activity of CYP27C1 might benefit lung cancer management. However, the specific role of CYP27C1 in cell signal transduction has not yet been identified, which could be one of the themes in future study.

Pacritinib is a selective JAK2 (wildtype and V617F) inhibitor, which can effectively inhibit the phosphorylation of downstream molecules (e.g., STAT3, STAT5, ERK1/2, Akt), thereby, hindering the signal transduction and leading to cell apoptosis [29,50]. JAK2/STAT3 is one of the crucial signaling pathways regulating multiple cellular activities, including cell proliferation, differentiation, and apoptosis. JAK2 initiates the phosphorylation process to each other upon the binding of ligands in the extracellular domains, and further triggers the phosphorylation and dimerization of STAT3, which allows its nuclear-translocation and exerts its function as transcription factors of downstream genes (e.g., p53, Cycling D1, cMyc, MCL-1) [51]. Studies found that p53 regulated the phosphorylation and DNA binding activity of STAT3 [52]. In addition, mutation and deletion in p53 was found along with constitutively activated STAT3 in cancer cells [52], which could further affect the sensitivity toward anticancer drugs [53]. Therefore, activating STAT3 might be the cause of hypersensitivity towards pacritinib in shCtrl-H1975 cells, harboring R273H-p53, whereas stable CYP27C1-knockdown could trigger a compensatory signaling pathway, e.g., IGF-1R/Akt/p53, to maintain cell survival. However, further study is necessary to reveal the specific role of CYP27C1 in the IGF-1R/Akt/p53 pathway.

Insulin-like growth factor (IGF) signaling is vital for cell activities and tissue development, and its dysregulation can lead to varied types of malignances [54], as well as the occurrence of anticancer drug resistance; therefore, targeting modulators in IGF signaling axis is promising in neoplastic management [24]. Recent studies found abundant crosstalk between the IGF-1R/Akt/mTORC1 signaling pathway and p53, which serves as a potent tumor suppressor in response to aberrant cell signal transduction induced by multiple external stress [23]. However, the roles of wildtype p53 are distinct from its mutants, some of which were identified as tumor promoter that aggravated the progression of malignances [55,56]. It is confirmed that p53-R273H was one of the mutants that exerted opposite roles to wildtype, which promotes cancer cell migration and invasion [55,56,57]. In this study, we found that the level of p53-wildtype significantly decreased in stable CYP27C1-knockdown A549 cells, whereas p53-R273H was upregulated in stable CYP27C1-knockdown H1975 cells, which could interpret the aggressive phenotype in stable CYP27C1-knockdown cell lines, including enhanced colony formation, accelerated cell proliferation, and cell migration. Moreover, p53-R273H exacerbates the resistance to tyrosine kinase inhibitors against mutated EGFR [58]. Here, to further study the underlying mechanism of the impaired potency of PPP, a selective IGF-1R inhibitor, we detected the level of phosphorylated Akt, total Akt, and p53 in stable CYP27C1-knockdown cells. The result indicated that CYP27C1 contributed to the sustention of the inhibitory effect of PPP.

Even though there is no evidence of direct physical interaction between CYP27C1 and any molecule in IGF-1R/Akt/p53 signaling pathway, the correlation between the expression level of CYP27C1, the protein level of Akt, and the level of p53 (wild-type and R273H mutant) were, for the first time, ascertained in this study. It revealed that CYP27C1 participated in regulation of tumor progression, and exerted function in maintaining the inhibitory effect of PPP via the IGF-1R/Akt/p53 signaling pathway.

## 4. Materials and Methods

### 4.1. Chemicals and Anticancer Agents

All the chemicals employed in this study were purchased from Sinopharm Chemical Reagent Co., Ltd. (Shanghai, China). Anticancer agents, vinorelbine ditartrate (S4269), picropodophyllin (S7668), pacritinib (S8057), and SKLB610 (S6526) were obtained from Selleck Chemicals (Houston, TX, USA).

### 4.2. Oligonucleotides and Plasmids

Cloning vector, pLKO.1 TRC (Cat. #10878), was obtained from Addgene (Watertown, MA, USA). Oligonucleotide synthesis and gene sequencing were conducted by IGE BIOTECHNOLOGY LTD (Guangzhou, China). Short-hairpin RNA targeting human CYP27C1 (5′-GGCCATTTATTCTGGAGAAGT-3′) were incorporated into pLKO.1 TRC cloning vector following the protocol provided by the manufacturer. Primers for real-time quantitative polymerase chain reaction (RT-qPCR) detecting *CYP27C1* (forward primer, 5′-CCAGAAGTGCAGCAGACGGTGTAC-3′; reverse primer, 5′-CTGGATCACGACGAGGTGAATCTC-3′), and the plasmid for CYP27C1 overexpression (pcDNA4-His/Max B-CYP27C1) were synthesized by IGE BIOTECHNOLOGY LTD.

### 4.3. Cell Culture and Cell Lines Establishment

All cell lines were purchased from the American Type Culture Collection (ATCC) (Rockville, MD, USA). Human bronchial epithelial BEAS-2B cells were maintained in the previously described culturing condition [59]. Human lung cancer cell lines (A549, H1299, H1975, H358, H460, and 95D) were cultured in complete growth medium containing 10% fetal bovine serum (Gibco, New York, NY, USA) and 1% penicillin-streptomycin (Gibco, New York, NY, USA), at 37 degrees Celsius in an atmosphere of 5% CO_2_/95% air. 

For the establishment of stable CYP27C1-knockdown A549, H1975 cell lines, and stable CYP27C1-overexpressed H460 cell line, plasmids (pLKO.1 TRC-shCYP27C1, pcDNA4-His/Max B-CYP27C1) were transfected into cells, respectively, followed by cell selection using 2 μg/mL of puromycin or 400 μg/mL of zeocin (Thermo Fisher Scientific, Waltham, MA, USA).

### 4.4. RNA Extraction, Reverse Transcription, and RT-qPCR

TRIzol Reagent (Thermo Fisher Scientific, Waltham, MA, USA) was applied to extract total RNA from the cells. GoScript™ Reverse Transcription System (Promega, Madison, WI, USA) was utilized to reverse-transcribe RNA to cDNA following the recommended protocol.

The relative expression level of *CYP27C1* in cells was assessed by real-time quantitative polymerase chain reaction (RT-qPCR), which was conducted on an ABI7500 Real-time PCR System (Applied Biosystems, Foster City, CA, USA), and analyzed with the comparative C_T_ method (2 ^−∆∆CT^ method).

### 4.5. Protein Extraction and Immunoblot Analysis

Cells were rinsed with phosphate buffered saline twice, and scratched from the surface of the culturing dishes and then lysed by prepared RIPA lysis buffer containing protease inhibitors and phosphatase inhibitors. A Bradford assay was performed to quantify the extracted total protein. Prepared protein samples were loaded for sodium dodecyl sulfate polyacrylamide gel electrophoresis (SDS-PAGE) using the steady current at 10 mA for each stacking gel, and 20 mA for each resolving gel. Resolved protein samples were transferred onto polyvinylidene difluoride (PVDF) membrane under constant voltage at 90 V for 105 min in an ice-cold water bath. The PVDF membrane was then blocked with skim milk (0.5%, *w*/*v*). As previously described, diluted primary antibodies targeting CYP27C1 (1:800, GTX45438, Gene Tex, Irvine, CA, USA), beta-actin (1:, A5441, Sigma Aldrich, St. Louis, MO, USA), phospho-tyrosine (1:2000, #9416, Cell Signaling Technology, Danvers, MA, USA), ERK1/2 (1:1000, #9102, Cell Signaling Technology), phosphorylated ERK1/2 Thr202/Tyr204 (1:2000, #4370, Cell Signaling Technology), Akt (1:1000, #4691, Cell Signaling Technology), phospho-Akt Ser473 (1:1000, #9271, Cell Signaling Technology), and p53 (1: 1000, sc-6243, Santa Cruz Biotechnology) were applied to probe the protein on PVDF membrane at four degrees Celsius overnight, following by the reaction with secondary anti-mouse or anti-rabbit antibodies at room temperature for two hours. Targeted proteins were detected using Enhanced Chemiluminescence Detection Reagents (GE Healthcare, Boston, MA, USA) and capture by Chemiluminescent Imaging System (Tanon 5200, Shanghai, China) [60]. Grey scales were analyzed by Gel-Pro ANALYZER.

### 4.6. Determination of the Effects of CYP27C1 on Cell Proliferation

Stable CYP27C1-knockdown A549 and H1975 cells, stable CYP27C1-overexpressed H460 cells, and the corresponding control cells were seeded in 96-well plates. Cell viability was tested by a 3-(4,5-dimethylthiazol-2-yl)-5-(3-carboxymethoxyphenyl)-2-(4-sulfophenyl)-2H-tetrazolium (MTS) assay at 12, 24, 36, 48, 60, 72, 84, and 96 h after cell seeding, following the manufacturer’s protocol (Promega, Madison, WI, USA), and the absorbance was measured by a microplate photometer (Thermo Fisher Scientific, Waltham, MA, USA) at 492 nm.

### 4.7. Establishment of In Vivo Xenograft Mouse Model

Athymic nude mice (BALB/c) were purchased from Beijing Vital River Laboratory Animal Technology Co., Ltd., and were maintained in a “specific pathogen-free” environment with free access to food and water. All animal experiments were conducted following the guideline approved by the Shantou University Medical College Institutional Animal Care and Use Committee. The ethical code is SUMC2019-321. Ten 6-week-old mice were divided into two groups, and injected subcutaneously with stable CYP27C1-knockdown H1975 cells and control cells (4 × 10^6^ cells in 200 μL of RPMI1640 basic medium) into the armpit of right forelimb, respectively. Tumors were formed nine days after injection, of which the greatest longitudinal diameter (*l*) and greatest transverse diameter (*w*) were measured by caliper. The volume (*V*) of the tumor was calculated using the following formula:

*V* = (*l* × *w*^2^)/2

The mice were sacrificed on the twenty-seventh day after tumor cell injection. Tumor weight was obtained and analyzed by the Mann–Whitney test.

### 4.8. Cell Colony Formation Assay

A cell colony formation assay was carried out as described in previous study [61]. In detail, stable CYP27C1-knockdown A549 and H1975 cells and the corresponding control cells were seeded in 6-well plates with a density of 2000 cells per well. Cell colonies, formed after around 14 days, were fixed by methanol at room temperature for 10 min, followed by staining with crystal violet (0.5%, *w*/*v*) of for 10 min. The images of stained cell colonies were captured by a digital scanner (V370 Photo, EPSON, Long Beach, CA, USA). The stained cell colonies were then eluted by acetic acid (10%, *v*/*v*), and the absorbance of eluent was measured by a microplate photometer at 595 nm.

### 4.9. Cell Scratch (Wound Healing) Assay

To compare the migration ability of stable CYP27C1-knockdown A549, H1975 cells, and stable CYP27C1-overexpressed H460 cells with that of the corresponding control cells, a cell scratch assay was performed as previously described with minor modifications [59]. Briefly, cells were seeded in 12-well plates with a density of 2 × 10^5^ cells per well. Surface of the cells was scratched to generate a wound using a pipette tip when the cells were at 90–95% confluency. The wound healing process was monitored and capture using a phase-contrast microscope. The scratching wound area was analyzed by ImageJ software. Wound healing rate was the ratio of the difference between wound area at two time points (0 h and 24 h) over the initial scratching area (0 h).

### 4.10. IC_50_ Values Assessment

Cells were seeded at the density of 1 × 10^4^ cells per well in 96-well plates for 24 h before the indicated treatments. Cell viability was detected by naphthol blue black (NBB) staining assay after inhibitory treatments for 48 h or 72 h. In detail, culture wells were rinsed with phosphate buffered saline to remove the dead cells. Remaining attached cells were fixed with methyl aldehyde (4%, *v*/*v*), and were then stained by NBB (0.05%, *w*/*v*) for 30 min. Culturing wells were rinsed with MilliQ H_2_O three times to remove the redundant dye, and the stained cells were eluted by NaOH solution (50 mM). The absorbance of the eluent was measured by microplate photometer at 595 nm. IC_50_ values were analyzed with the equation of log(inhibitor) vs. response—Variable slope of nonlinear regression in GraphPad Prism 7.

### 4.11. Vinorelbine and Protein Kinase Inhibitors Sensitivity

Stable CYP27C1-knockdown A549 and H1975 cells, stable CYP27C1-overexpressed H460 cells, and the corresponding control cells were seeded in 24-well plates for 24 h, followed by treatments of vinorelbine (0, 1.25, 2.5, 5, 10, and 20 nM for A549 and H460; 0, 2.5, 5, 10, 20, and 40 nM for H1975) for 72 h. NBB assay was performed to detect cell viability as described above.

Stable CYP27C1-knockdown cells, and the corresponding control cells were treated with picropodophyllin (0.5 and 1 μM), pacritinib (1 and 2 μM), and SKLB610 (5 and 50 μM) after seeding for 24 h. Cell viability was detected by MTS assay at 12, 24, 36, 48, 60, 72, 84, and 96 h after cell seeding, or by real-time cell index obtained by the RTCA-S16 System (San Diego, CA, USA).

### 4.12. Statistical Analysis

Data in each section was obtained from at least three independent experiments. All of the statistical analyses, including unpaired student’s *t*-test, two-way ANOVA, nonlinear regression, Mann–Whitney test were conducted using GraphPad Prism 7.

## 5. Conclusions

In this study, we found that CYP27C1 was differentially expressed in human lung cancer cell lines compared to human normal bronchial epithelial BEAS-2B cells. The potency of vinorelbine and protein kinase inhibitors was aggressively impaired by CYP27C1-knockdown, and revealed that CYP27C1 was critical for vinorelbine or protein kinase inhibitors inducing cytotoxicity in human lung cancer cells. CYP27C1-knockdown regulated the signal transduction in the IGF-1R/Akt/p53 signaling pathway by altering the level of Akt, downregulating the level of p53-wildtype, or increasing the level of p53-R273H, resulting in aberrant cell proliferation, colony formation, and cell migration. Therefore, CYP27C1 plays an indispensable role in dictating the cellular sensitivity of human lung cancer cells towards anticancer agents.

## Figures and Tables

**Table 1:** The IC_50_ values of four anticancer agents in human lung cancer cells.

Cell Line	Time	IC_50_ Values (Mean ± SD)			
		Vinorelbine (nM)	Picropodophyllin (μM)	Pacritinib (μM)	SKLB610 (μM)
A549	48 h	1.89 ± 0.66	0.29 ± 0.14	2.73 ± 1.02	5.71 ± 0.73
	72 h	1.76 ± 0.60	0.31 ± 0.11	2.61 ± 0.54	5.68 ± 0.75
H1975	48 h	33.87 ± 0.12	0.45 ± 0.05	1.34 ± 0.45	N/A
	72 h	10.06 ± 0.70	0.44 ± 0.10	1.21 ± 0.30	N/A
H460	48 h	2.27 ± 0.50	0.40 ± 0.02	2.73 ± 0.44	18.35 ± 1.26
	72 h	1.60 ± 0.54	0.44 ± 0.07	3.70 ± 0.43	13.09 ± 1.12
H1299	48 h	N/A	0.38 ± 0.03	3.42 ± 0.53	8.78 ± 0.94
	72 h	N/A	0.29 ± 0.09	2.70 ± 0.43	6.41 ± 0.81

N/A: Not applicable.

## Data Availability

All the data presented in this study are available upon request from the corresponding author.

## Supplementary Materials

The following supporting information can be downloaded at: https://www.mdpi.com/article/10.3390/ijms23147853/s1.
