# Supplementary material for: Cytochrome P450 27C1 Level Dictates Lung Cancer Tumorigenicity and Sensitivity towards Multiple Anticancer Agents and Its Potential Interplay with the IGF-1R/Akt/p53 Signaling Pathway
Source: Int J Mol Sci. 2022 Jul 16;23(14):7853. doi: 10.3390/ijms23147853 (PMC9324654; doi:10.3390/ijms23147853)
Supplement: Supplementary file 1 [file ijms-23-07853-s001.zip › ijms-1766562-supplementary.pdf]

A

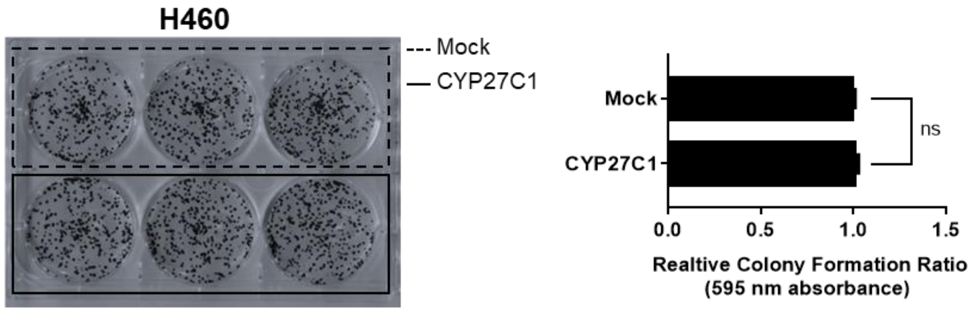

B

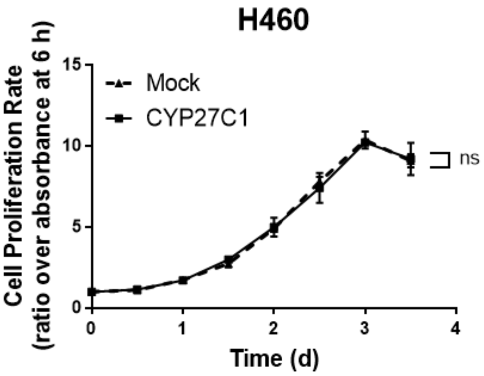

Supplementary Figure S1. Cell colony formation ability and proliferation of stable CYP27C1-overexpressed H460 cells. (**A**) Stable CYP27C1-overexpressed H460 cells and control cells were seeded in 6-well plates respectively. Culture medium was changed in every 3-4 days, cell colony formation was observed. After culturing for about 14 days, cell colonies were stained with crystal violet, and scanned. The absorbance of eluent was detected at 595 nm. Results were analyzed by unpaired two-tailed T test. n.s., $p > 0.05$. (**B**) Cell viability of Stable CYP27C1-overexpressed H460 cells and control cells was detected by MTS assay at different time point. Time-cell proliferation curves were plotted, and analyzed by Two-way ANOVA. n.s., $p > 0.05$.

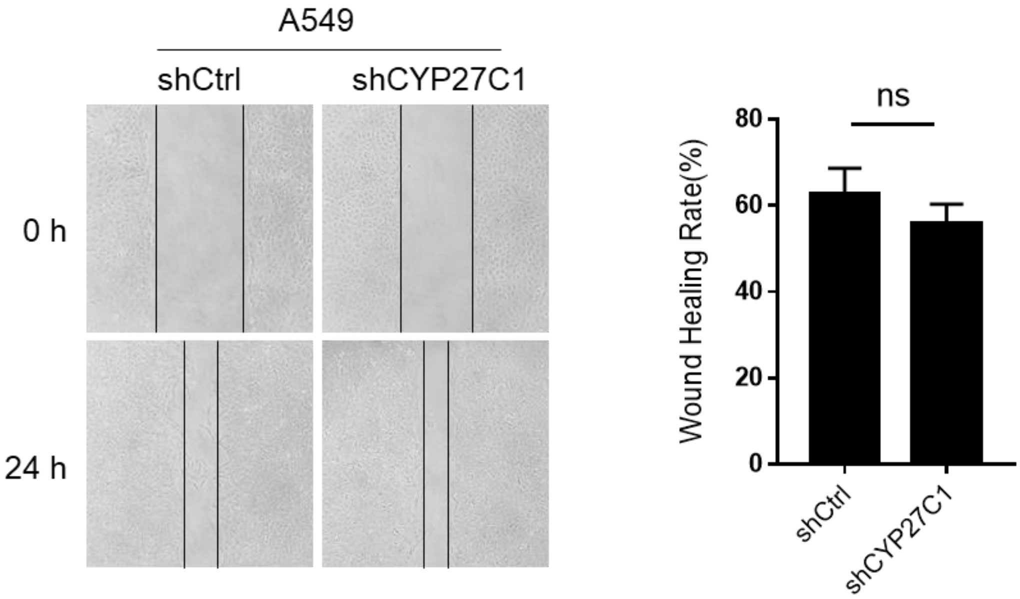

Supplementary Figure S2. Cell migration ability of stable CYP27C1-knockdown A549 cells. A549-shCYP27C1, and the corresponding control cells were seeded in 12-well plates for 24 hours before cell scratch assay. Wound areas were captured at 0, 24 hours after scratching, and analyzed by ImageJ software.

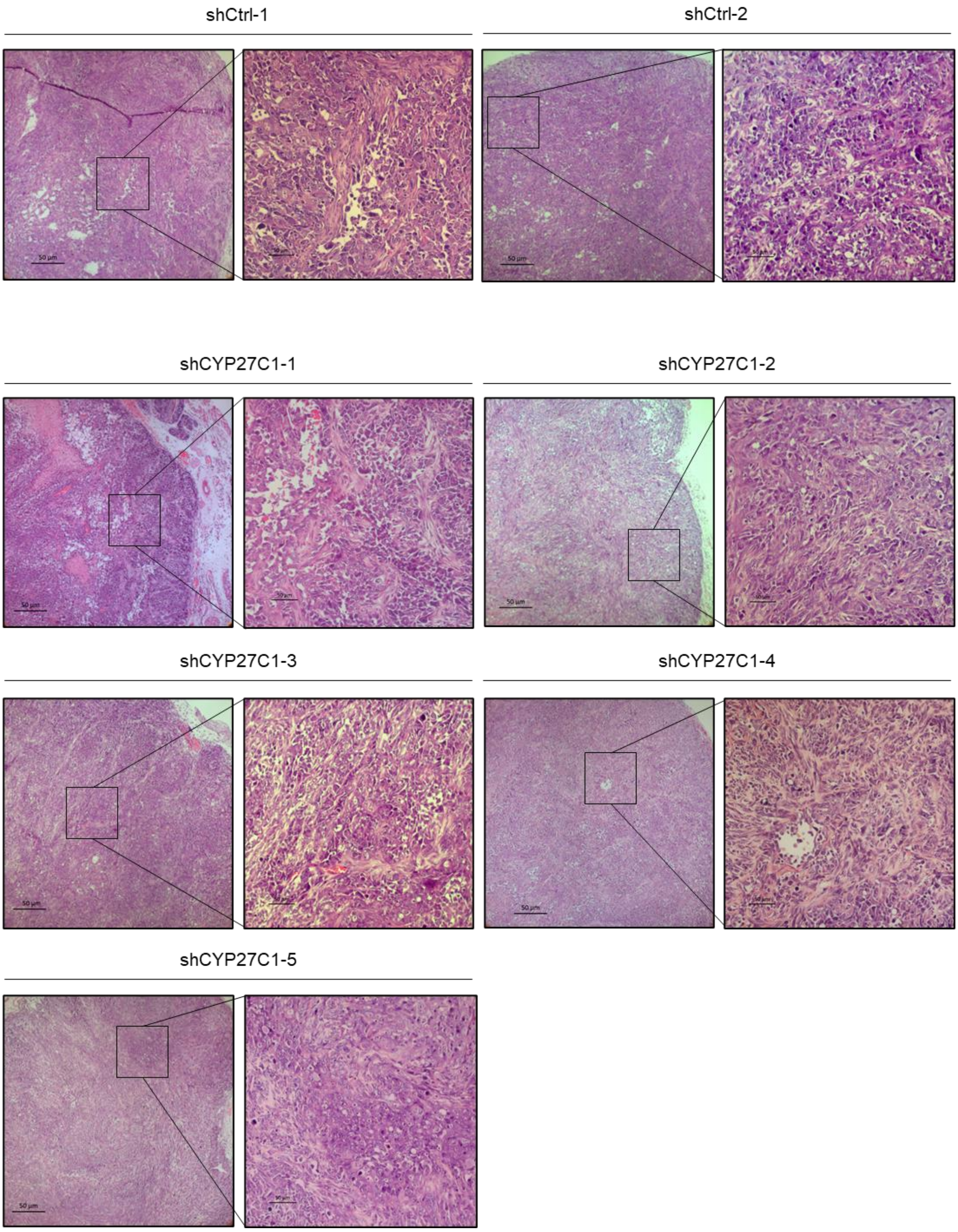

Supplementary Figure S3. Histology (hematoxylin-eosin staining) of xenograft tumor in nude mice model. Magnification: 50× (left panel); 200× (right panel).

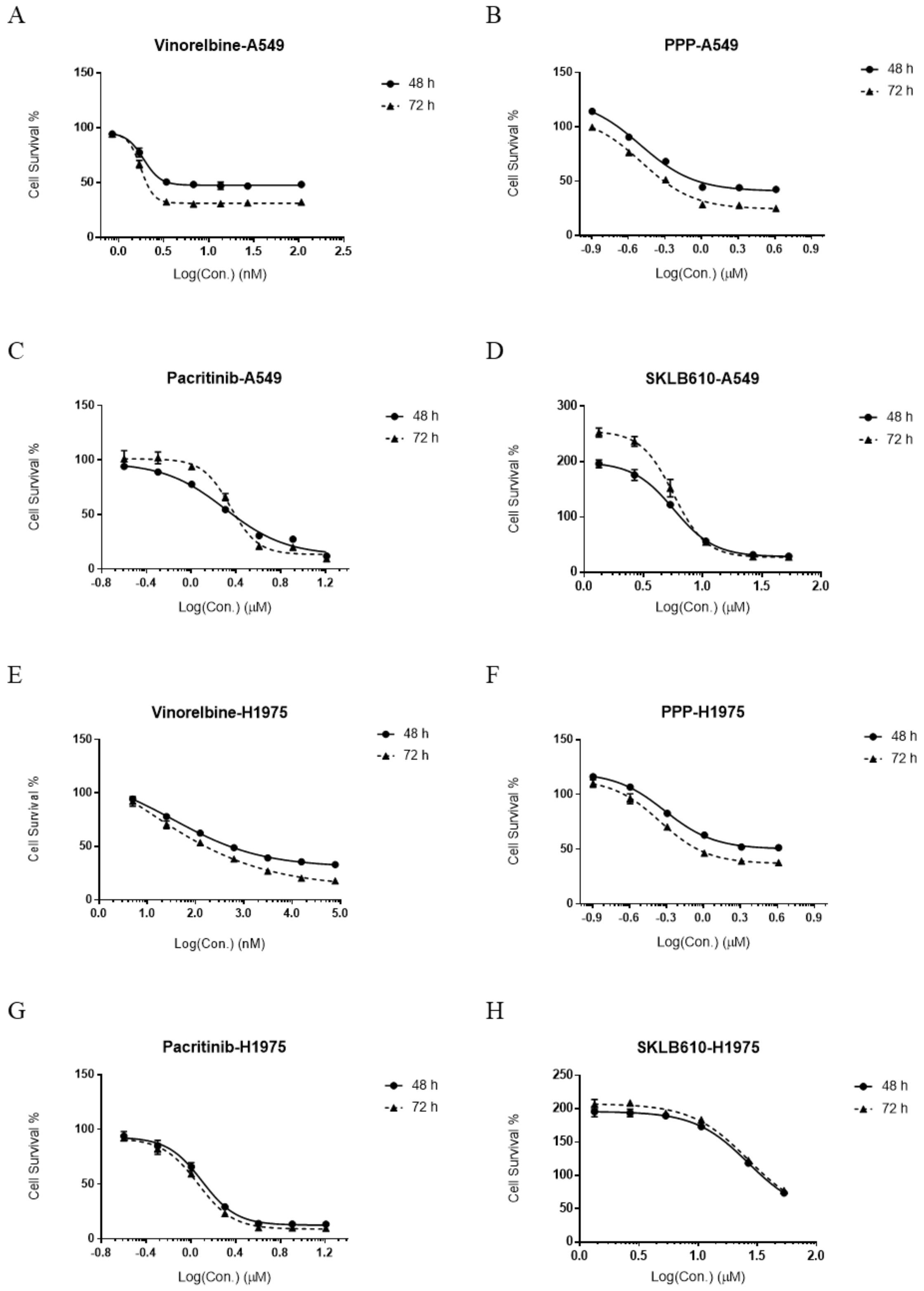

Supplementary Figure S4. Sensitivity toward anticancer agents in CYP27C1 differentially expressed human lung cancer cells.

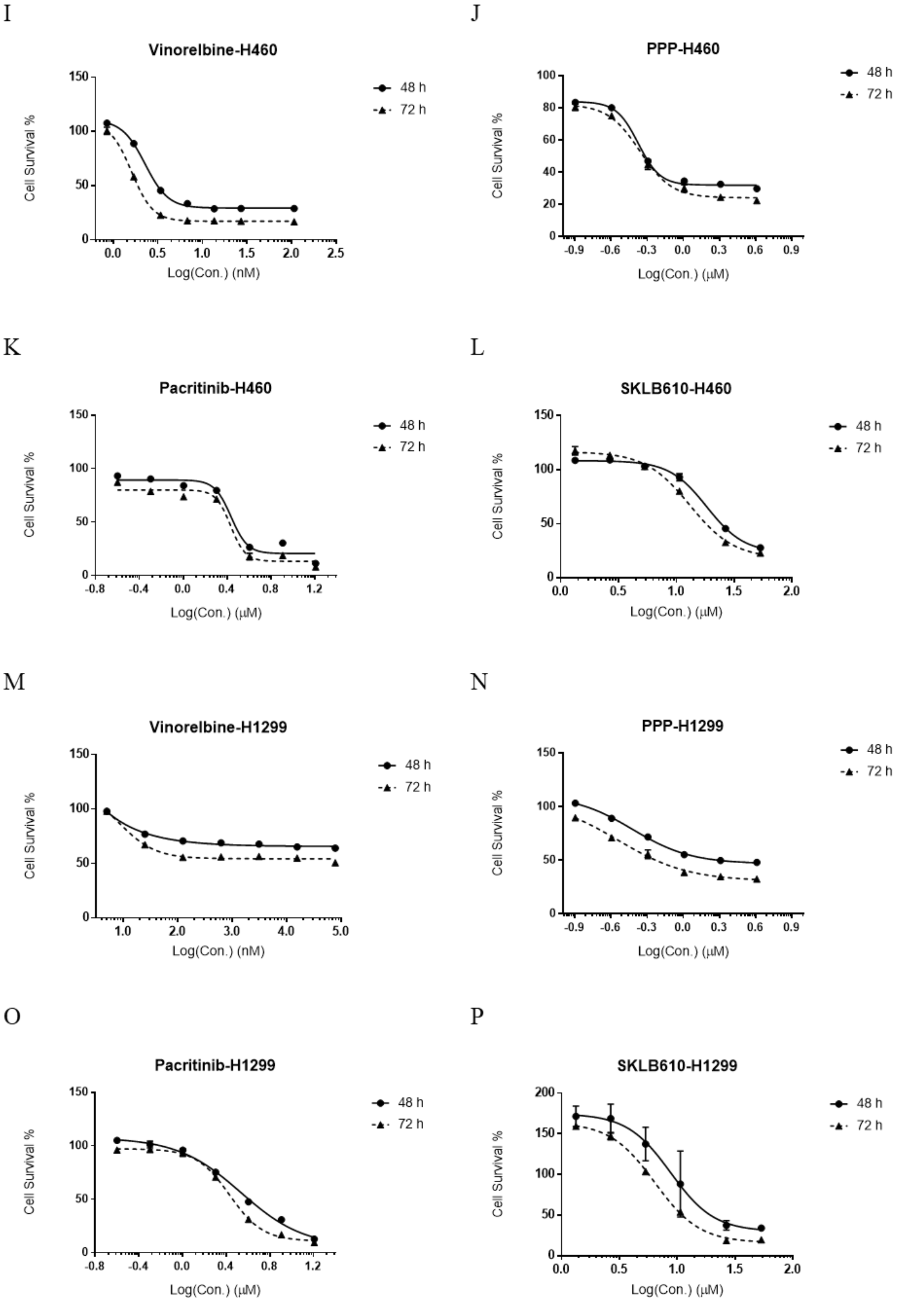

Supplementary Figure S4. Sensitivity toward anticancer agents in CYP27C1 differentially expressed human lung cancer cells.
